# Electronic Public Health Registry of Extensively Drug-Resistant Organisms, Illinois, USA

**DOI:** 10.3201/eid2110.150538

**Published:** 2015-10

**Authors:** William E. Trick, Michael Y. Lin, Robynn Cheng-Leidig, Mary Driscoll, Angela S. Tang, Wei Gao, Erica Runningdeer, M. Allison Arwady, Robert A. Weinstein

**Affiliations:** Rush University Medical Center, Chicago (W.E. Trick, M.Y. Lin, R.A. Weinstein);; Cook County Health and Hospitals System, Chicago, Illinois, USA (W.E. Trick, W. Gao, R.A. Weinstein);; Illinois Department of Public Health, Springfield, Illinois, USA (R. Cheng-Leidig, M. Driscoll, A.S. Tang, E. Runningdeer, M.A. Arwady)

**Keywords:** automated medical records system, drug resistance, microbial, registries, computers, bacteria, antimicrobial resistance, extensively drug-resistant organisms, Illinois, United States

## Abstract

In response to clusters of carbapenem-resistant *Enterobacteriaceae* (CRE) in Illinois, USA, the Illinois Department of Public Health and the Centers for Disease Control and Prevention Chicago Prevention Epicenter launched a statewide Web-based registry designed for bidirectional data exchange among health care facilities. CRE occurrences are entered and searchable in the system, enabling interfacility communication of patient information. For rapid notification of facilities, admission feeds are automated. During the first 12 months of implementation (November 1, 2013–October 31, 2014), 1,557 CRE reports (≈4.3/day) were submitted from 115 acute care hospitals, 5 long-term acute care hospitals, 46 long-term care facilities, and 7 reference laboratories. Guided by a state and local public health task force of infection prevention specialists and microbiologists and a nonprofit informatics entity, Illinois Department of Public Health deployed a statewide registry of extensively drug-resistant organisms. The legal, technical, and collaborative underpinnings of the system enable rapid incorporation of other emerging organisms.

## Abstract 2

This technology-based public health tool can facilitate detection of and communication about these bacteria.

The emergence of extensively drug-resistant organisms (XDROs) is a major public health problem because few or no effective antimicrobial drugs are available to treat infections caused by these bacteria (*1*). In the United States, carbapenem-resistant *Enterobacteriaceae* (CRE) are XDROs considered high priority for control (*2*–*4*), and regional clusters have been detected in Illinois (*5*,*6*) and elsewhere (*7*). Control of drug-resistant bacteria is possible (*8*,*9*) but requires a coordinated regional effort across the spectrum of health care facilities (*10*,*11*). Failure to control spread of antimicrobial drug–resistant bacteria hinders medical care at a growing number of facilities by creating hazardous opportunities for untreatable infections during aggressive medical interventions (*12*), such as immunosuppressive therapies and device insertions, or during common endoscopic procedures (*2*,*13*).

To combat CRE, the Centers for Disease Control and Prevention (CDC) recommends a “Detect and Protect” strategy: detect CRE patients through systematic surveillance and protect patients by preventing transmission of CRE through application of appropriate infection control precautions when such patients enter a health care facility (*14*). Because a patient is often cared for at multiple health care facilities (*15*,*16*), ensuring that information follows a patient is challenging: survivors of prolonged intensive care unit (ICU) treatment go through a median of 4 facility care transitions, including non–acute care facilities, within 1 year (*16*). To improve the effectiveness of the Detect and Protect strategy, information needs to be shared routinely among facilities, but information sharing often is suboptimal (*4*,*17*). Innovative tools to automate information sharing have been developed (*18*) but have focused on hospitals; comprehensive systems are needed that extend beyond acute care hospitals and encompass large geographic regions.

Before 2013, the Illinois Department of Public Health (IDPH) had limited information about the epidemiology of CRE. A CDC-funded surveillance activity (REALM project) (*5*), consisting of point prevalence studies of CRE carriage among ICU patients in Chicago acute care hospitals and all patients in long-term acute care hospitals (LTACHs) was ongoing; however, prevalence data were limited to Chicago and did not include patients outside the ICU or in long-term care facilities (LTCFs). The first function of the XDRO registry was to provide a mechanism for standardized reporting of CRE carrier patients from all health care facilities throughout the state.

In November 2013, IDPH launched a public health informatics tool called the XDRO registry (http://www.xdro.org), designed to facilitate information exchange throughout health care facilities in Illinois. The first function of the registry was to provide a mechanism for standardized reporting of patients in whom CRE was detected. The registry, an electronic platform for CRE information exchange, receives reports (Figure 1) of CRE in accordance with a state-enacted surveillance rule (Table 1) and centrally stores patient-specific CRE information (Figure 2). The registry was developed through collaboration among public health agencies (federal, state, city, and local), informatics specialists, infection control professionals, microbiologists, and academic researchers. We report our experience designing and implementing the Illinois XDRO registry.

**Figure 1:**
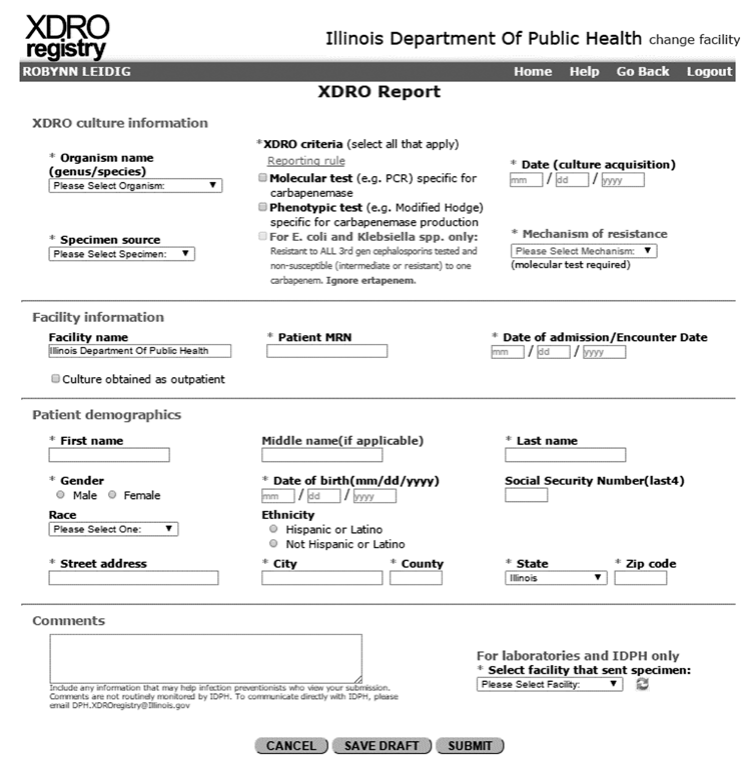
Display of Illinois XDRO registry’s submission page. Completion of 1 report is contained within this single page. Asterisk indicates required fields. Field names in gray font (i.e., *Escherichia coli* and *Klebsiella* spp. criterion and mechanism of resistance) are conditioned on prior responses, organism name and XDRO criteria, respectively. The field “For laboratories and IDPH only” is not visible for other users; this field enables public health and reference laboratories to input isolates for facilities that have not submitted a report. XDRO, extensively drug-resistant organism.

**Table 1:** CRE definition used in the XDRO registry, Illinois, USA*

*Enterobacteriaceae* (e.g., *Escherichia coli*, *Klebsiella* spp., *Enterobacter* spp., *Proteus* spp., *Citrobacter* spp. *Serratia* spp., *Morganella* spp., or *Providentia* spp.) with 1 of the following laboratory results:
1. Molecular test (e.g., PCR) specific for carbapenemase.
2. Phenotypic test (e.g., modified Hodge) specific for carbapenemase production.
3. For *E. coli* and *Klebsiella* spp. only: nonsusceptible (intermediate or resistant) to 1 of the following carbapenems (doripenem, meropenem, or imipenem) AND resistant to ALL of the following third-generation cephalosporins tested (ceftriaxone, cefotaxime, and ceftazidime). Note: ignore ertapenem for this definition.

*As of November 1, 2013. CRE, carbapenem-resistant *Enterobacteriaceae*; XDRO, extensively drug-resistant organism.

**Figure 2:**
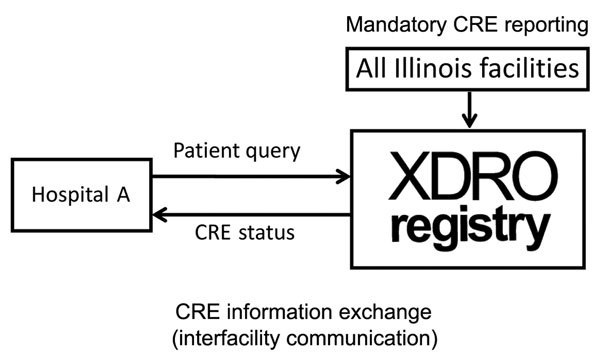
Conceptual framework of the XDRO registry, Illinois, USA. XDRO, extensively drug-resistant organism.

## Registry Development

### Partnership Development and Key Participants

In response to the emergence of CRE in Illinois (*5*), we conceptualized and began developing the XDRO registry in early 2011; the registry went live November 1, 2013. The registry was conceived of and developed by a partnership among public health, academia, infection preventionists (i.e., infection prevention specialists in healthcare facilities), and a nonprofit public health informatics entity. Given its jurisdiction over reporting of communicable diseases, IDPH sponsored the registry. The CDC Chicago Prevention Epicenter provided expertise in designing and implementing the registry. Medical Research Analytics and Informatics Alliance (MRAIA), a 501(c)3 entity designated as an agent of IDPH for public health reporting and related activities, developed and hosted the Web interface, database, and software application for automating alerts. IDPH expanded the Chicago Department of Public Health’s CRE advisory group to a statewide task force to gather input from relevant disciplines.

### Design Rationale: CRE Reporting Considerations

#### Mandated versus Voluntary Reporting

One early decision centered on whether the registry should be a voluntary or a mandated reporting system. A voluntary system had the advantage of relatively rapid deployment but would have had incomplete reporting, particularly from facilities not already actively engaged in submitting case reports to IDPH. Instead, motivated by anticipated improvements in reporting adherence, we pursued a mandated approach that required a change in Illinois’ public health rules. Although time consuming, the public vetting process provided transparency and a valuable opportunity for feedback from and acceptance by the infection prevention community. The registry rule was proposed in November 2012 and, after public comment, was finalized in September 2013 with an implementation date of November 1, 2013 (*19*). During the rule-making process, we spent time engaging partners (especially the existing statewide health care–associated infections advisory council), designing and testing the website, and establishing database encryption.

Prior studies in Illinois demonstrated that CRE disproportionately affected chronically ill patients in acute care hospitals (both short- and long-term) and certain LTCFs that cared for mechanically ventilated patients (*5*,*20*). Few patients in the community without health care exposure were colonized with CRE (*20*). On the basis of this evidence, IDPH mandated reporting from all acute care hospitals (short- and long-term) and LTCFs. Laboratories also were required to report CRE, consistent with IDPH’s requirement for reporting other infections; this requirement increased reporting of CRE for 2 reasons: 1) laboratories could report for facilities that were not used to or had limited resources for public health reporting, such as LTCFs, and 2) laboratories could report for health care settings not covered by the mandate, such as outpatient clinics. For reference laboratories outside of Illinois, the health care facility must report the isolate.

#### Creating the CRE Definition

We created the Illinois CRE registry definition in accordance with the interim CDC definition proposed in the 2012 CDC CRE Toolkit (*14*). In general, CRE definitions are designed to identify the subset of CRE that produces carbapenemases (rather than noncarbapenemase mechanisms of resistance, such as membrane permeability changes) because carbapenemase-producing *Enterobacteriaceae* have a greater propensity to cause outbreaks. Because the definition does not have perfect specificity for carbapenemase-producing *Enterobacteriaceae*, we retained the term CRE. Although the priority of many surveillance efforts is high sensitivity (i.e., capturing most true cases), we preferred specificity (i.e., few false positives) because the XDRO registry shares information among providers with the intent of implementing infection control precautions, which might negatively affect some patients. Thus, we chose to restrict the antimicrobial drug susceptibility testing criterion (Table 1) to *Escherichia coli* and *Klebsiella* species, because other *Enterobacteriaceae* can have intrinsic imipenem nonsusceptibility (*14*). Other reportable *Enterobacteriaceae*, such as *Enterobacter* species, require a more specific microbiologic test (such as a molecular or phenotypic confirmatory test) (*21*).

We also decided to make the first CRE-positive culture per patient stay reportable to the registry, regardless of whether the patient previously had been reported. We accept multiple reports for a patient to provide accepting facilities with information about when the patient was last reported as a CRE carrier. There is no time limit on patient retention in the registry because duration of CRE carriage is unknown and studies suggest prolonged and possibly indefinite carriage (*22*–*24*).

### Design Rationale: Information Exchange Considerations

**Figure 3:**
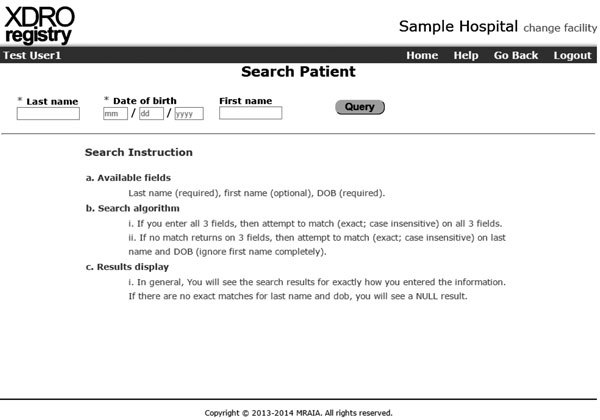
Illinois XDRO registry query page. The patient's last name and date of birth are required to execute a search. CRE, carbapenem-resistant *Enterobacteriaceae*; XDRO, extensively drug-resistant organism.

Before the registry, the process for sharing patient-specific CRE information among health care providers during direct facility-to-facility patient transfer was by written or verbal communication; such communication was inconsistent. Even with complete communication among facilities during direct facility-to-facility transfer, such communication would affect only the ≈20% of interfacility patient sharing that is direct (*15*). The XDRO registry improves interfacility sharing of CRE information across serial health care facility visits by enabling authorized personnel at any Illinois health care facility to query whether a patient of unknown CRE status has been reported to the registry (Figure 3).

#### Patient Privacy

Sharing patient-level CRE status raised patient privacy concerns. Patients routinely sign consent for their information to be shared for direct facility-to-facility patient transfers, but no explicit authorization is granted for sharing information across disconnected visits (*25*). However, infection control information is unique in that sharing information among health care facilities is expected to occur to prevent spread of antimicrobial drug–resistant organisms to persons at the receiving facility, and electronic alert systems to identify such patients are promoted as a means of protecting patients. After consultation with public health legal counsel at IDPH and CDC, we interpreted the following Health Insurance Portability and Accountability Act provision under General Public Health Activities as authority to build a public health registry and enable interfacility communication to prevent XDRO transmission: “The Privacy Rule permits covered entities to disclose protected health information, without authorization, to public health authorities who are legally authorized to receive such reports for the purpose of preventing or controlling disease, injury, or disability. This would include, for example, the reporting of a disease or injury; reporting vital events, such as births or deaths; and conducting public health surveillance, investigations, or interventions” (*26*). Previous effective interventions support the idea that successful control of XDRO transmission requires active communication among facilities (*8*,*27*).

#### Patient Identifier

Exchange of patient information across facilities requires a reliable patient identifier. In Illinois, as in most states, no established master patient identifier existed. We could not rely on Social Security numbers because many patients do not report a number and, for privacy reasons, some hospitals prohibit staff from viewing Social Security numbers. Thus, we combined patient name and date of birth to create an identifier (*28*), which simplified querying patients because such information is routinely accessible to hospital staff.

For automated bidirectional exchange of registry data among health care facilities, we developed software that creates a 1-way hash. We use a deterministic match on the hashed identification (ID); that is, we require exact replication of the name plus date of birth (*28*). We create 3 hashes that all include date of birth but vary on completeness of name, as follows: 1) full first + last names, 2) first initial of first name + full last name, or 3) full last name alone. Regardless of which query matched, we display the patient’s first and last name to the user. When a patient match is reported by using a manual query, the registry provides a disclaimer that the infection preventionist needs to verify the patient’s CRE status. Verification options include comparing the patient’s registry and admission address, asking the patient or family member whether the patient had been in the reporting health care facility at the time of CRE occurrence, or retesting the patient for CRE carriage.

#### XDRO Registry Access

Nearly all communicable diseases in Illinois are reported to the Illinois National Electronic Disease Surveillance System (I-NEDSS). Access to I-NEDSS is controlled through a password-protected Web portal. We paired the XDRO registry with the I-NEDSS application through the same Web portal, such that authorized I-NEDSS users automatically are given access to the registry. All user permissions are managed through IDPH’s existing security infrastructure; the registry stores authentication and activity logs for audit. To protect patient privacy, and because CRE information requires verification by infection control staff, we limit registry access to personnel authorized to electronically submit reportable diseases (i.e., usually infection preventionists at hospitals, designated reporters in free-standing laboratories, and selected persons at LTCFs).

### Technical Infrastructure

The XDRO registry is built on a Web-based platform. The home page (http://www.xdro.org) is publically accessible and displays language for the rule, training materials, frequently asked questions, and a password-protected link to access the registry. The registry is housed on co-located servers (Prominic Inc., Champaign, IL, USA) and managed by personnel at IDPH and the Medical Research Analytics and Informatics Alliance.

## Challenges and Solutions

We have encountered numerous challenges during development of the XDRO registry (Table 2). We highlight several challenges that other public health agencies interested in building a similar registry are likely to encounter.

**Table 2:** Selected challenges encountered and solutions offered during development of a statewide XDRO registry, Illinois, USA

Challenge	Solution
Legal and regulatory: sharing patient CRE information without explicit informed consent	Public health rule written to authorize reporting/sharing of CRE information, as allowed under HIPAA 45 CFR 164.512(b)
Technical, security	
Securely maintain username/password permissions	IDPH maintains permissions through existing portal infrastructure in parallel with the I-NEDSS application. User table synchronized with XDRO registry permissions.
Electronic laboratory reporting of CRE results	Not implemented; standardized values not defined for all CRE criteria. Custom codes need to be created. Reconciliation between electronic and manual reports will require development.
Data accuracy	
Susceptibility criterion exclusive to *Klebsiella* *pneumoniae* and *Escherichia* *coli*. Selected inappropriately for other organisms	To prevent users from including other species for this “susceptibility criterion,” this criterion could not be selected unless *K. pneumoniae* or *E. coli* were chosen as the organism.
No master patient identifier available	Combinations of patient last name, first name, and date of birth used as an identifier (Figure 3). Disclaimer to hospital staff to confirm matched patient queries.
No universal health care facility identifier available	We use existing IDPH facility codes. LTCFs that do not have I-NEDSS access do not have an identifier and are encouraged to enroll in I-NEDSS.
CRE events are entered without systematic validation of data entry	Web entry form has logic embedded to minimize data entry errors. A microbiologic validation of a subset of CRE isolates will be performed in 2015.
Single users reporting for multiple facilities	Facility drop-down list created for users who report from multiple facilities. User–facility relationships managed by email request to the registry and human verification.
Non-*Enterobacteriaceae* entered through free-text option	The free text option was removed. *Pseudomonas* spp. were the most common non-*Enterobacteriaceae* entered.
Work flow	
Manual query function is time consuming	Manual querying is most appropriate for facilities with few admissions (e.g., LTCFs). IDPH is developing an automated query system for large facilities.
Administratively linked, geographically distinct facilities assigned same code	Request facilities to submit reports as distinct facilities.
CRE definition changes	CDC has proposed new criteria for identifying CRE, which requires updating website design and rules.
Health departments want to edit cases	Developed after the launch and for now restricted to a few users at the state health department who understand when edits and entries are appropriate.
Reference laboratories report CRE events for health care facilities	Each reference laboratory designates a reporter for the registry. Reports linked to individual facilities through a customized drop-down list during submission process.

CDC, Centers for Disease Control and Prevention; CRE, carbapenem-resistant *Enterobacteriaceae*; HIPAA, Health Insurance Portability and Accountability Act; IDPH, Illinois Department of Public Health; I-NEDSS, Illinois Notifiable Electronic Surveillance System; LTCF, long-term care facility; XDRO, extensively drug-resistant organism.

### Need for a Standardized Unique Health Care Facility Identifier

Accurate identification of facilities is critical for detecting regional clusters of XDROs, assigning reports from reference laboratories to the correct institution, and enabling interfacility communication of alerts; however, no comprehensive unique identification system existed for health care facilities. Although all facilities have a National Provider Identifier (NPI) number, that number can be associated with billing units within facilities, so that a single hospital can have multiple associated NPIs. We elected to use an existing set of identifiers known as “site codes” maintained by IDPH to link I-NEDSS users to their institution (IDPH controls access to their secure portal). An advantage of using IDPH site codes was that users with security clearance for reporting to I-NEDSS were automatically granted XDRO registry access, linked to their established I-NEDSS facility.

Although most acute care hospitals had unique site codes, LTCFs historically had reported to I-NEDSS through local health departments without a site code. The absence of LTCF site codes presented a challenge because these facilities often use reference laboratories to detect CRE. Because we could not list unregistered facilities, we had to rely on free text, which complicates cluster detection. We permit reference laboratories to create a list of “favorites” to minimize the variability inherent to free text entries. Also, we actively encourage LTCFs to register in the I-NEDSS system.

Another challenge to using site codes was that sometimes geographically distinct facilities shared a site code. The primary reason for shared site codes was that some health care systems elected to use a single site code across geographically distinct facilities for public health reporting. Because most other reportable diseases are tracked primarily by patient address rather than reporting facility, facility location usually is unnecessary. To correct this problem, IDPH actively reached out to such facilities to encourage assignment of unique site codes.

### Lack of Routine Validation of CRE Events

A limitation of our system is that, for several reasons, reported CRE isolates are not routinely validated. However, the goal of the registry is to facilitate interfacility communication, and in current practice, patients are deemed CRE-positive by infection preventionists and laboratory personnel on the basis of laboratory criteria. Our long-term vision is to automate reporting with minimal manual entry (i.e., automated electronic laboratory reporting of CRE), which would occur before microbiologic validation of CRE. Given the relatively large number of CRE isolates in Illinois, it was not feasible for a central laboratory to validate every isolate. Instead of routine validation of each isolate, we ask each laboratory to submit a sample of CRE isolates (5 per laboratory) to a reference laboratory to validate organism identification, susceptibility testing, and carbapenemase production.

### Time-Consuming Manual Queries

The XDRO registry is designed to enable infection preventionists to query the registry to determine whether newly admitted patients carry CRE. In practice, because manually searching the registry for each admitted patient is time consuming, comprehensive manual queries are likely only for facilities with few patient admissions (e.g., LTCFs or LTACHs). Large facilities can query a subset of high-risk patients.

To realize the fundamental goal of rapid notification of infection preventionists, we are piloting automated CRE alerting from the registry. Health care facilities participating in automated alerting electronically transmit an encrypted list of patient admissions that periodically are matched to the registry (e.g., hourly or daily). When a match is found between an admitted patient and the registry, authorized personnel at the admitting health care facility receive an email directing them to log into the registry and view their alert history; the email contains no patient identifiers.

### Registry Updates and Maintenance

We rapidly address XDRO registry concerns through close collaboration between IDPH, CDC Prevention Epicenter investigators, and the informatics team. During development and the initial year, we scheduled weekly conference calls to discuss user concerns, make website modifications, and plan educational outreach. In the second year, we scheduled meetings every 2 weeks. Users contact IDPH with registry concerns through a dedicated email address or by phone; the contact information is available through the XDRO website. The Illinois CRE task force of local experts in microbiology, infection prevention, and public health provides ongoing counsel. Training webinars were conducted at the time of registry launch, and refresher webinars continue periodically; training materials (including recorded webinar sessions) are accessible through the XDRO registry website.

CDC continues to evaluate the CRE definition as new data and diagnostic tests become available (*29*). As definitions evolve, the registry must accommodate changes. Modifications will require substantial revisions to the website, reeducation of users, and changes in rules for automated alerts. Changing definitions also will make it difficult to follow trends in the number of CRE cases reported. The registry platform enables expansion to other organisms; in particular, the security, search functionality, report distribution to public health, and automated notifications will scale well. However, we will have to develop new reporting rules, web pages, and data tables for additional organisms.

## First Year of Registry

To describe XDRO registry activity, we analyzed the first full year of de-identified data (November 1, 2013–October 31, 2014). For patient and organism descriptive analysis, data were deduplicated at the patient level so that only the first report per patient was retained for analysis.

During the initial 12 months, 1,557 reports were submitted to the XDRO registry, an average of 4.3 per day. These reports contained 1,095 unique patients (1.4 entries for each individual patient). Data were entered from 173 unique facilities: 115 (64%) of 181 registered acute care hospitals, 5 (56%) of 9 LTACHs, 46 LTCFs, and 7 reference laboratories. For some health systems, a single user accessed the registry for several facilities. Because each user’s logon is linked to a single primary facility, we have incomplete counting of the number of facilities that have reported to the system.

The median age of reported patients was 64 years (interquartile range 54–75 years; range 11–>90 years); 52% of patients were female. For ≈43% of unique patients, a mechanism of resistance was known: of those, most common was *K. pneumoniae*–producing carbapenemase (KPC) (461 [98%] of 472), followed by New Delhi metallo-β-lactamase (NDM) (11 [2%] of 472). Most KPC-producing *Enterobacteriaceae* were *Klebsiella* species (84%); most NDM-producing *Enterobacteriaceae* were *E. coli* (85%). Specimens collected were from urine (45%); rectum (19%); wound (12%); sputum (12%); blood (6%); and body fluid, tissue, or other (6%). On average, slightly more than 30 unique facilities queried the registry each month.

We assessed registry use through a paper-based survey of hospitals and LTACHs in the Chicago metro area that participated in a separate public health surveillance project (*6*). During 1 survey period (January–July 2014), hospitals designated an infection preventionist to answer a 15-item written questionnaire about CRE control, of which 3 questions focused on the facility’s use of the registry. Twenty-one (88%) of 24 acute care hospitals and all 7 LTACHs responded. Of the respondents, 86% of acute care hospitals and 100% of LTACHs reported having at least 1 person registered to access the registry. Fifty-five percent of hospitals and 43% of LTACHs had queried the status of a CRE-unknown patient. Most acute care hospitals did not routinely query (59%) or queried occasionally (32%); none queried every admitted patient. In contrast, 2 (29%) of 7 LTACHs queried all patients on admission. Ninety-six percent of hospitals expressed interest in automated CRE alerts.

## Future Directions

Future efforts to improve the registry fall primarily under 2 domains: facility alerts and cluster detection for public health. To automate CRE alerting, we are exploring data interfaces between health care facilities and the registry in a way that can scale to many facilities. For example, we are working with a surveillance software vendor common among health care facilities in Illinois. For health care facilities without a vendor solution, we install our hashed ID software system locally so that no protected health information is transmitted to the registry.

IDPH receives weekly automated reports that are manually reviewed to detect clusters or identify reports of uncommon resistance mechanisms. We are exploring enhanced regional situational awareness of potential CRE clusters by using statistical programs (e.g., SaTScan, http://www.satscan.org) that detect spatial or space-time CRE clusters. For example, by geocoding all defined health care facilities and following facility-specific CRE counts across time, individual facilities or groups of facilities can be surveyed for statistically significant changes in CRE reporting. Clusters can be defined on the basis of geographic distance (e.g., all health care facilities within a certain radius) or can be defined within patient sharing networks (e.g., health care facilities that commonly share patients, even if geographically disparate). Such clusters can trigger additional investigation by public health epidemiologists to determine whether an outbreak is occurring.

## Conclusions

CRE surveillance and interfacility communication are recommended for regional infection control but difficult to achieve in practice. We formed a unique partnership among public health, academic investigators, and a nonprofit entity to develop an informatics solution to these challenges. The XDRO registry is an example of a technology-based public health tool that can facilitate CRE detection and communication.
